# Granulomatosis with Polyangiitis Discovered Because of Repeated Upper Eyelid Swelling

**DOI:** 10.3390/medicina60091555

**Published:** 2024-09-23

**Authors:** Suguru Nakagawa, Kiyohito Totsuka, Shinichiro Kagami, Yohei Nomoto

**Affiliations:** 1 Department of Ophthalmology, Asahi General Hospital, Chiba 289-2511, Japan; 2 Department of Ophthalmology, Saitama Medical Center, Jichi Medical University, Saitama 330-0834, Japan; 3 Department of Allergy and Clinical Immunology, Asahi General Hospital, Chiba 289-2511, Japan

**Keywords:** granulomatosis with polyangiitis, antineutrophil cytoplasmic antibodies, eyelid swelling, episcleritis, periocular cellulitis, corticosteroids

## Abstract

*Background and objectives*: The initial symptom that triggers granulomatosis with polyangiitis (GPA) diagnosis is rarely ocular. We describe a case with a single ocular lesion identified as probable GPA due to proteinase 3 (PR3)-antineutrophil cytoplasmic antibody (ANCA)-positivity according to the diagnostic criteria of the Ministry of Health in Japan; the lesion repeatedly worsened. *Materials and methods*: A 25-year-old female visited the Department of Ophthalmology, Asahi General Hospital, with upper eyelid swelling and conjunctival and episcleral hyperemia of the left eye. Both hordeolum and eyelid cellulitis were suspected, as the condition was resistant to treatment with antibiotic eye drops. Episcleritis was suspected due to localized hyperemia in the upper part of the eye. Upon treatment with antibacterial agents and steroid eye drops, the swelling and the hyperemia repeatedly worsened every week. *Results*: Blood samples were positive for PR3-ANCA, and GPA with an isolated ocular lesion was considered. After oral steroid treatment, the patient had no recurrence for 4 years. There was no systemic involvement in the upper respiratory tract, lungs, or kidneys. *Conclusions*: Diagnosing GPA with ocular symptoms as initial manifestations is challenging. GPA should be considered in treatment-resistant eyelid, orbital, and episcleral lesions, even at a young age.

## 1. Introduction

Granulomatosis with polyangiitis (GPA) is a systemic disease characterized by necrotizing vasculitis of small arteries and veins [1]. It includes a triad of necrotizing granulomas of the upper and lower respiratory tracts, systemic vasculitis, and necrotizing glomerulonephritis. GPA is a vasculitis syndrome, the pathogenesis of which involves antineutrophil cytoplasmic antibodies (ANCAs). The initial symptoms that trigger the diagnosis are rarely ocular, and the age range of GPA is epidemiologically widely distributed, with a peak age range of 64–75 years [2,3]. Fifteen percent of patients with GPA have ocular and orbital symptoms at the initial presentation [4,5,6].

Herein, we report the case of a 25-year-old woman with a single ocular lesion diagnosed with GPA (probable) owing to prominent upper eyelid swelling with repeated exacerbations resistant to antibacterial and steroid eye drops and positive proteinase 3 (PR3)-ANCA.

## 2. Case Report

**Figure 1:**
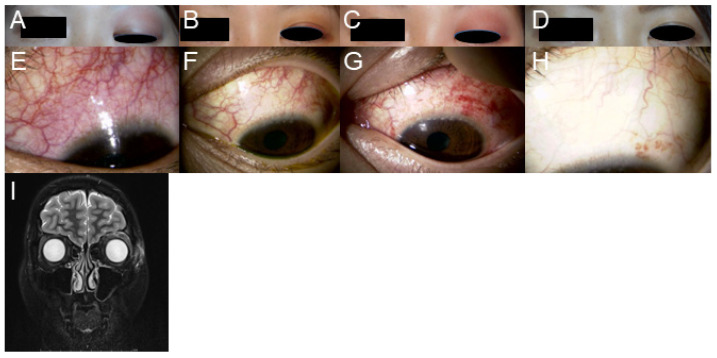
(**A**). Eyelid findings at the initial examination. Upper eyelid swelling is observed. (**B**). Eyelid findings 3 days after the initial examination. Upper eyelid swelling improved with antibacterial and steroid eye drops. (**C**). Eyelid findings 1 month after the initial examination. Upper eyelid swelling worsened again. (**D**). Eyelid photograph 4 months after systematic steroid treatment. The eyelid swelling resolved. (**E**). Anterior segment of the eye at the initial examination. Episcleritis is observed in the upper eye. (**F**). Anterior segment of the eye 3 days after the initial examination. Hyperemia in the upper eye improved with antibacterial and steroid eye drops. (**G**). Anterior segment of the eye 1 month after the initial examination. Episcleritis exacerbated again. (**H**). Anterior segment of the eye 4 months after steroid treatment. No hyperemia was observed, and episcleritis resolved. (**I**). Orbital magnetic resonance image (short TI inversion recovery) at the initial examination. Edematous signal changes are observed in the left eyelid and orbicular muscle. Periorbital cellulitis was suspected.

A 25-year-old Japanese female visited her local doctor complaining of left eye pain and redness for 1 month. As episcleritis was suspected, she was prescribed antibacterial eye drops and steroid eye drops; however, no improvement was observed for one month. She visited the Department of Ophthalmology, Asahi General Hospital, in February 2017, presenting with upper eyelid swelling and conjunctival and episcleral hyperemia in the left eye (Figure 1A, upper and middle panels). The patient had been wearing a 1-month disposable soft contact lens (SCL) since she was 15 years of age. She had no food or drug allergies. No abnormalities were observed in the posterior ocular regions. Her decimal-corrected visual acuity was (1.2) in the right eye and (1.2) in the left eye. The intraocular pressure was 15 mmHg in the right eye and 18 mmHg in the left eye. Hertel exophthalmometry revealed no ocular protrusion in the right 16 mm/left 16–17 mm. Schirmer’s method revealed decreased lacrimal secretions of 2 mm on the right side and 3 mm on the left side. Orbital magnetic resonance imaging revealed edematous signal changes in the left eyelid and periorbital skin (Figure 1A, bottom panel). We suspected hordeum, periorbital cellulitis, and episcleritis. The patient was treated with 1.5% levofloxacin eye drops and 0.1% betamethasone eye drops; however, the eyelid swelling worsened and improved markedly every week (Figure 1B,C). Throughout the follow-up period, there were no inflammatory findings in the anterior chamber, and the vitreous body and fundus did not show any inflammatory complications such as vitreous opacities or vasculitis. The corrected visual acuity and intraocular pressure were within the normal range during the follow-up period. We measured the intraocular pressure once per one or two weeks during the topical steroid therapy.

After a blood sample was positive for PR3-ANCA, the patient was referred to the Department of Collagen Disease, where she was diagnosed with GPA (probable) with an ocular lesion alone according to the diagnostic criteria of the Health and Welfare Ministry in Japan. Otolaryngological examination, electrocardiogram, and chest computed tomography revealed no abnormalities. Treatment with prednisolone (PSL; 40 mg/day) was initiated 1 month after she first visited our hospital. Dramatic improvements in hyperemia and eyelid swelling were observed immediately after treatment initiation. The dose was thus decreased by 10 mg every week, then by 2.5 mg every week up to 10 mg, and then by 1 mg every month from 10 mg. After PSL was reduced to 6 mg/day (7 months after the patient’s first visit to our clinic), PR3-ANCA became negative. Thereafter, the PSL dose was gradually reduced, 1 mg at a time, to 4 mg at 15 months after the initial visit, 3 mg at 2 years and 2 months, 2 mg at 2 years and 7 months, and 1 mg at 3 years; eventually, PSL was discontinued at 4 years and 2 months. During this time, the patient remained negative for PR3-ANCA and had no recurrent ocular symptoms.

## 3. Discussion

We encountered a case of GPA with an ocular lesion alone. In this case, periocular cellulitis, episcleritis, and PR3-ANCA positivity were observed. The patient was considered to have GPA (probable) because of ocular symptoms and positive PR3-ANCA, according to the GPA diagnostic criteria [7,8,9] of the Japanese Ministry of Health, Labor and Welfare. In addition, this case met the classification criteria for GPA according to the American College of Rheumatology/European Alliance for Rheumatology [10] with positivity for PR3-ANCA (5 points) and was diagnosed as GPA with a cumulative score of 5 points.

GPA is epidemiologically widely distributed, with an annual incidence of 2.1 to 14.4 per million people [11,12]. Ocular symptoms [11,13,14] occur in 40–60% of GPA cases [11,15], and the initial symptom is an ocular lesion in approximately 15% of cases (n = 158) [4,5,6].

Hoffmann et al. reported that scleritis/episcleritis, conjunctivitis, ocular protrusion, and dacryocystitis were present in 6%, 5%, 1%, and 1% of patients with GPA, respectively [6]. Moin et al. reported that scleritis/episcleritis, conjunctivitis, ocular protrusion, and dacryocystitis were present in 49.2%, 8.8%, 33.1%, and 21.5% of patients with GPA who were PR3-ANCA positive, respectively [16]. Ocular lesions rarely precede upper and lower respiratory tract symptoms, and GPA is relatively rarely detected because of ocular symptoms. GPA with an initial symptom of ocular lesions takes the longest time to diagnose, with an average of 14 months [17].

ANCA is an important determinant of the onset of GPA and is usually detectable [11,18], as in our case. According to statistics, PR3-ANCA is positive in 60–75% of cases and myeloperoxidase (MPO)-ANCA in 20–30%, while less than 5% of cases are ANCA-negative [11,18]. The intrinsic and extrinsic factors combine to reduce resistance to neutrophil autoantigens PR3 and MPO and then activate autoreactive T and B cells, which produce ANCA, an autoantibody that promotes the accumulation of neutrophils in the microvasculature and adhesion to vascular endothelium. Activated neutrophils produce reactive oxygen species, proteases, and neutrophil extracellular traps, which induce programmed cell death. Furthermore, the ANCA antigens produced are presented to effector T cells for antigen recognition, causing the release of inflammatory cytokines and further mobilization of effector leukocytes [11,18]. These responses result in inflammatory damage to endothelial cells and other tissues in GPA, but the exact molecular biological mechanisms remain unknown.

Although the onset of GPA during the 20–30 age range, as in this case, is rare, GPA has been reported in children [3,19]. GPA responds well to steroid therapy, and the ophthalmologic prognosis is reported to be relatively good with early treatment [20,21]. In the present case, the patient had a healthy ocular condition, and the ophthalmologic prognosis was good with early treatment with no recurrence for four years.

A potential limitation in this case was that the resolution by the systemic administration of steroids can occur not only in cases of GPA but also in cellulitis, scleritis, and various inflammatory conditions.

## 4. Conclusions

In the present case, a 25-year-old healthy woman presented with swelling of the upper eyelid of the left eye and hyperemia of the conjunctiva and sclera; we suspected hordeum, periocular cellulitis, and episcleritis. She was treated with antibacterial and steroid eye drops; however, her eyelid swelling worsened markedly and improved every week. Subsequently, a thorough systemic examination led to the diagnosis of GPA, which allowed us to begin treatment before the onset of systemic symptoms. GPA should be considered for treatment-resistant eyelid, orbital, and scleral lesions, even in young patients. The initial manifestation of ocular lesions requires the longest time to diagnose GPA. Therefore, awareness of GPA is essential for ophthalmologists to make the correct diagnosis and prescribe the appropriate treatment.

## Data Availability

The datasets generated and/or analyzed during the current study are available from the corresponding author upon reasonable request.

## References

[1] Zeek P.M. (1953). Periarteritis nodosa and other forms of necrotizing angiitis. N. Engl. J. Med..

[2] Ntatsaki E., Watts R.A., Scott D.G. (2010). Epidemiology of ANCA-associated vasculitis. Rheum. Dis. Clin..

[3] Kubaisi B., Samra K.A., Foster C.S. (2016). Granulomatosis with polyangiitis (Wegener’s disease): An updated review of ocular disease manifestations. Intractable Rare Dis. Res..

[4] Rasmussen N. (2001). Management of the ear, nose, and throat manifestations of Wegener granulomatosis: An otorhinolaryngologist’s perspective. Curr. Opin. Rheumatol..

[5] Macarie S.S., Kadar A. (2020). Eye involvement in ANCA positive vasculitis. Rom. J. Ophthalmol..

[6] Hoffman G.S., Leavitt R.Y., Kerr G.S., Fauci A.S. (1992). The treatment of Wegener’s granulomatosis with glucocorticoids and methotrexate. Arthritis Rheum..

[7] Sada K., Yamamura M., Harigai M., Fujii T., Dobashi H., Takasaki Y., Ito S., Yamada H., Wada T., Hirahashi J. (2014). Classification and characteristics of Japanese patients with antineutrophil cytoplasmic antibody-associated vasculitis in a nationwide, prospective, inception cohort study. Arthritis Res. Ther..

[8] Sada K.-e., Yamamura M., Harigai M., Fujii T., Arimura Y., Makino H., Research Committee on Intractable Vasculitides, the Ministry of Health, Labour and Welfare of Japan (2015). Issues associated with the Ministry of Health, Labour and Welfare diagnostic criteria for antineutrophil cytoplasmic antibody-associated vasculitides: Reclassification of patients in the prospective cohort study of Remission Induction Therapy in Japanese patients with ANCA-associated vasculitides according to the MHLW criteria. Mod. Rheumatol..

[9] Sada K.-e., Nagasaka K., Kaname S., Higuchi T., Furuta S., Nanki T., Tsuboi N., Amano K., Dobashi H., Hiromura K. (2024). Evaluation of Ministry of Health, Labour and Welfare diagnostic criteria for antineutrophil cytoplasmic antibody–associated vasculitis compared to ACR/EULAR 2022 classification criteria. Mod. Rheumatol..

[10] Robson J.C., Grayson P.C., Ponte C., Suppiah R., Craven A., Judge A., Khalid S., Hutchings A., Luqmani R.A., Watts R.A. (2022). 2022 American College of Rheumatology/European Alliance of Associations for Rheumatology classification criteria for granulomatosis with polyangiitis. Arthritis Rheumatol..

[11] Mei L., Wang L., Yan H. (2023). Updates of ocular involvement in granulomatosis with polyangiitis. Graefe’s Arch. Clin. Exp. Ophthalmol..

[12] Mohammad A.J. (2020). An update on the epidemiology of ANCA-associated vasculitis. Rheumatology.

[13] Sfiniadaki E., Tsiara I., Theodossiadis P., Chatziralli I. (2019). Ocular manifestations of granulomatosis with polyangiitis: A review of the literature. Ophthalmol. Ther..

[14] Byszewska A., Skrzypiec I., Rymarz A., Niemczyk S., Rękas M. (2023). Ocular involvement of granulomatosis with polyangiitis. J. Clin. Med..

[15] Ismailova D., Abramova J., Novikov P., Grusha Y. (2018). Clinical features of different orbital manifestations of granulomatosis with polyangiitis. Graefe’s Arch. Clin. Exp. Ophthalmol..

[16] Moin K.A., Yeakle M.M., Parrill A.M., Garofalo V.A., Tsiyer A.R., Bishev D., Gala D., Fogel J., Hatsis A.J., Wickas T.D. (2023). Ocular and orbital manifestations of granulomatosis with polyangiitis: A systematic review of published cases. Rom. J. Ophthalmol..

[17] Srouji I., Andrews P., Edwards C., Lund V. (2007). Patterns of presentation and diagnosis of patients with Wegener’s granulomatosis: ENT aspects. J. Laryngol. Otol..

[18] Kitching A.R., Anders H.-J., Basu N., Brouwer E., Gordon J., Jayne D.R., Kullman J., Lyons P.A., Merkel P.A., Savage C.O.S. (2020). ANCA-associated vasculitis. Nat. Rev. Dis. Primers.

[19] Gajic-Veljic M., Nikolic M., Peco-Antic A., Bogdanovic R., Andrejevic S., Bonaci-Nikolic B. (2013). Granulomatosis with polyangiitis (Wegener’s granulomatosis) in children: Report of three cases with cutaneous manifestations and literature review. Pediatr. Dermatol..

[20] Brubaker R., Font R.L., Shepherd E.M. (1971). Granulomatous sclerouveitis: Regression of ocular lesions with cyclophosphamide and prednisone. Arch. Ophthalmol..

[21] Bullen C.L., Liesegang T.J., McDonald T.J., DeRemee R.A. (1983). Ocular complications of Wegener’s granulomatosis. Ophthalmology.

